# Assessing the histidine-rich protein 2/3 gene deletion in *Plasmodium falciparum* isolates from Burkina Faso

**DOI:** 10.1186/s12936-023-04796-9

**Published:** 2023-11-29

**Authors:** Casimire Wendlamita Tarama, Harouna Soré, Mafama Siribié, Siaka Débé, Réné Kinda, Wendyam Gérard Nonkani, Farida Tiendrebeogo, Winnie Bantango, Kassoum Yira, Esther Yéri Hien, Moussa Wandaogo Guelbéogo, Yves Traoré, Didier Ménard, Adama Gansané

**Affiliations:** 1 Centre National de Recherche et de Formation sur le Paludisme, Ouagadougou, Burkina Faso; 2 Université Joseph KI-ZERBO, Ouagadougou, Burkina Faso; 3 Institut Pasteur, Université Paris Cité, Malaria Genetic and Resistance Unit, INSERM U1201, 75015 Paris, France; 4 Institut Pasteur, Université Paris Cité, Malaria Parasite Biology and Vaccines, F-75015 Paris, France; 5 Institute of Parasitology and Tropical Diseases, Université de Strasbourg, UR7292 Dynamics of Host-Pathogen Interactions, 67000 Strasbourg, France; 6 Laboratory of Parasitology and Medical Mycology, CHU Strasbourg, 67000 Strasbourg, France

**Keywords:** Malaria, *Plasmodium falciparum*, *hrp2*, *hrp3*, *Burkina Faso*

## Abstract

**Background:**

Dual *hrp2/hrp3* genes deletions in *P. falciparum* isolates are increasingly reported in malaria-endemic countries and can produce false negative RDT results leading to inadequate case management. Data on the frequency of *hrp2/hrp3* deleted parasites are rarely available and it has become necessary to investigate the issue in Burkina Faso.

**Methods:**

*Plasmodium falciparum-*positive dried blood spots were collected during a cross-sectional household survey of the malaria asymptomatic children from Orodara, Gaoua, and Banfora. Amplicons from the target regions (exon 2 of *hrp2* and *hrp3* genes) were generated using multiplexed nested PCR and sequenced according to Illumina’s MiSeq protocol.

**Results:**

A total of 251 microscopically positive parasite isolates were sequenced to detect *hrp2* and *hrp3* gene deletions. The proportion of RDTs negative cases among microscopy positive slides was 12.7% (32/251). The highest prevalence of negative RDTs was found in Orodara 14.3% (5/35), followed by Gaoua 13.1%(24/183), and Banfora 9.1% (3/33). The study found that 95.6% of the parasite isolates were wild type *hrp2/ hrp3* while 4.4% (11/251) had a single *hrp2* deletion. Of the 11 *hrp2* deletion samples, 2 samples were RDT negative (mean parasitaemia was 83 parasites/ μL) while 9 samples were RDT positive with a mean parasitaemia of 520 parasites /μL (CI95%: 192–1239). The highest frequency of *hrp2* deletion 4/35 (11.4%) was found in Orodara, while it was similar in the other two sites (< 3.5%). No single deletion of the *hrp3* or dual deletion *hrp2/3* gene was detected in this study.

**Conclusion:**

These results demonstrate that *P. falciparum* isolates lacking *hrp2* genes are present in 4.4% of samples obtained from the asymptomatic children population in three sites in Burkina Faso. These parasites are circulating and causing malaria, but they are also still detectable by HRP2-based RTDs due to the presence of the intact *pfhrp3* gene.

## Background

Malaria is a major public health concern in Burkina Faso. More than 12 million cases of malaria have been reported, among which 605,504 cases of severe malaria and 4,355 deaths in 2021 [1]. The World Health Organization (WHO) recommends parasitological confirmation before treatment for malaria with quality-assured microscopy or RDTs [2]. Early and accurate diagnosis of malaria is thus becoming an essential pillar in the effective management and control of malaria. Microscopic examination is the oldest laboratory method and, although it is recognized as the gold standard, it has certain limitations, including a shortage of qualified microscopists, adequate infrastructure, and logistical resources, particularly in remote, resource-poor areas [3, 4]. To overcome the shortage of well-trained technicians in microscopic diagnosis in many areas of sub-Saharan Africa, the WHO recommends to use rapid diagnostic tests (RDTs) as an alternative method for diagnosing malaria [2]. RDTs are the primary tools used in the field, require little training, are easy and quick to perform and allow diagnosis even in remote areas prior to the prescription of anti-malarial drugs. RDTs are lateral flow immunochromatography tests that detect parasite specific antigens in the blood [7]. The most commonly used RDTs are those detecting either species-specific *Plasmodium falciparum* histidine-rich protein 2 (PfHRP2) or *P. falciparum* lactate dehydrogenase (PfLDH), or both *P. falciparum*-specific antigens and pan-specific antigens (aldolase or pLDH) [5, 6]. The genes encoding the HRP2 and HRP3 proteins are located on chromosomes 8 and 13, respectively, in the *P. falciparum* genome [8]. Interestingly, due to conserved repetitive epitopes between the two antigens, some monoclonal antibodies directed against HRP2 cross-react with HRP3 [9, 10]. The HRP2 protein is abundant in the blood of malaria patients. It is specifically expressed by *P. falciparum* and is an excellent target for diagnosis. HRP2-targeted RDTs are used in most countries in sub-Saharan Africa where *P. falciparum* malaria is prevalent [5, 11]. According to the WHO treatment guidelines, all suspected cases of malaria in Burkina Faso are confirmed by RDTs or microscopy before treatment [2]. Since 2018, confirmation of suspected cases of malaria in Burkina Faso using HRP2-targeting RDTs has risen from 88.9% to 99.4% in 2021 [1].

However, the performance of HRP2-based RDTs continues to be threatened by the emergence of *P. falciparum* parasite strains with a dual deletion in the *hrp2* and *hrp3* genes. Parasites with deletions of both these genes can cause false negative results on HRP2-based RDTs [12]. *Plasmodium falciparum* field isolates from the Amazon region of Peru were first shown to be negative with HRP2-based RDTs due to the deletion of *hrp2* and *hrp3,* and their respective flanking genes [13]. Since then, a growing number of reports from Latin America and Asia have identified varying proportions of parasites with *hrp2* deletion [14, 15]. Parasites lacking either or both *hrp2* and *hrp3* have also been reported to occur at varying frequencies in East Africa, West Africa, and Central Africa [12, 16–21]. To date, the proportion of *P. falciparum* strains with either or both *hrp2* and *hrp3* gene deletions has ranged from 62% to 0.4% in selected studies in sub-Saharan Africa [22]. Across Africa, the highest prevalence of both *hrp2* and *hrp3* gene deletions has been reported in some regions of Eritrea, with frequencies of 80.8% and 92.3%, respectively [23]. The emergence of parasites lacking *hrp2* and *hrp3* gene remains a major threat to public health due to the widespread use of PfHRP2-detecting *P. falciparum*–only RDTs. Consequently, the current solution to the diagnostic problem associated with the circulation of *P. falciparum* parasites lacking *hrp2* and *hrp3* is to first estimate their prevalence to decide whether it is necessary to replace the RDT. The WHO currently recommends switching to more effective alternative non-HRP2 RDTs when the prevalence of *pfhp2*-deleted parasites causing false negative RDTs exceeds 5% in symptomatic patients [24].

The presence or absence of *P. falciparum* parasites carrying the *hrp2/3* gene deletion has not been assessed in Burkina Faso. To address this lack of information, a cross-sectional household survey of children was conducted to provide useful information to guide malaria control strategies for the judicious selection of RDTs used to detect malaria infection.

The aim of this study was to provide some preliminary evidence for the existence of *P. falciparum* lacking the *pfhrp2* gene which may affect the accuracy of malaria diagnosis by HRP2-based RDTs in Burkina Faso.

## Methods

### Study site and design

**Fig. 1:**
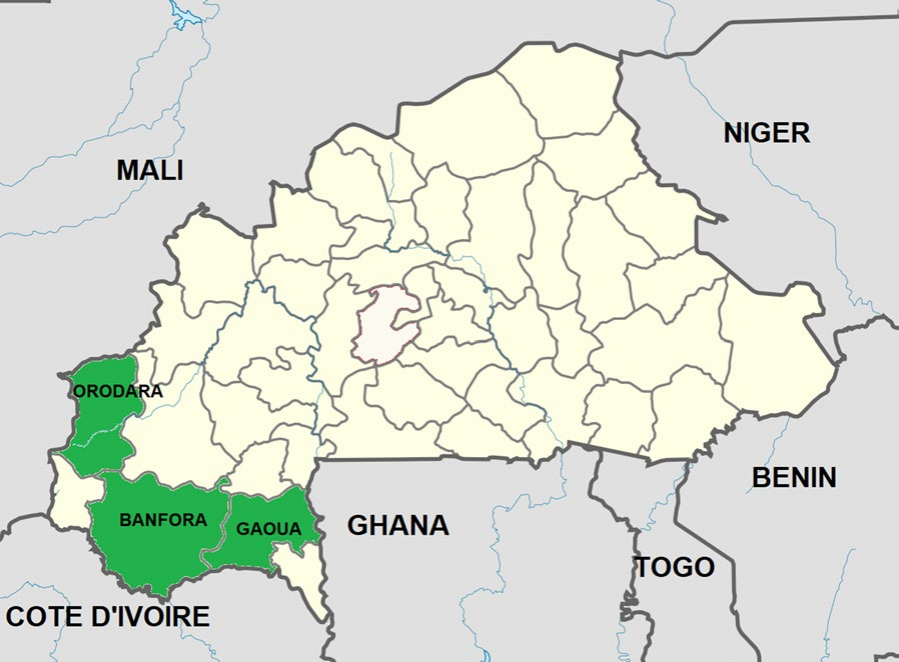
Location of the study sites, Burkina Faso, 2022–2023

The study was conducted in the health districts of Banfora, Orodara, and Gaoua located and western and southern parts of Burkina Faso (Fig. 1). In these regions, the burden of malaria is permanent, and the mortality rate remains the highest in the country. The entomological inoculation rate (EIR) ranges from 0.91 to 2.35 infective bites/person/day [25, 26] A cross-sectional household survey on the asymptomatic children was carried out at the start of the rainy season in July 2022, the peak period for malaria transmission. Using a standard systematic sampling approach clustered by sector[27], 190 households with a child aged 6–59 months and/or 190 children aged 5–10 years in each study district were selected. A total of 380 children were enrolled in 10 randomly selected villages in each health district. This study was part of a larger research project that aimed to investigate the prevalence of asymptomatic malaria using rapid diagnostic tests. Light microscopy-positive *P falciparum* samples were used to assess deletion of the histidine-rich protein 2/3 gene.

### Laboratory procedures

#### Samples collection and processing for malaria screening

Blood samples collected aseptically from each participant by finger prick was analysed under a light microscope to detect the presence of parasites. Blood samples were also tested by RDT (AdvDx Malaria Pf Rapid Malaria Ag Detection Test # 00-DKM-RK-MALADX-004–025, Advy Chemical Pvt Ltd, India) and used to prepare 903TM Whatman TM (LASEC 10530151 Rev.AC, USA) five-spot blood card which stored at room temperature in a sealed plastic bag containing a desiccant. All field samples (slides, and dried blood spots) were then transported to the CNRFP laboratory in Ouagadougou, Burkina Faso. Dried blood spots (DBS) were sent to the Institute of Parasitology and Tropical Diseases in Strasbourg and the Institut Pasteur in Paris, France, for molecular analyses.

This WHO prequalified (reference number: PQDx 0345-101-00) RDTs were performed in the field and interpreted by well-trained nurses in accordance with the manufacturer's instructions. Thick and thin blood smears were read by two independent level 1 certified malaria microscopists from the Clinical Laboratory Service according to CNRFP standard protocols. Slides were re-read by a third independent microscopist if the two readers disagreed on the species, presence or absence of malaria parasites, or if parasite densities differed by > 25%. The final value for parasite density was the mean of the two closest readings. Parasites were counted on the thick film and the parasite density was calculated using the formula: number of parasites counted × 8000/number of leukocytes counted. If no parasites were detected in at least 200 fields, the slide was considered negative.

#### Parasite DNA extraction, PCR analysis and DNA sequencing

Each dried blood spot was sterile-cut and placed in an Eppendorf tube. Parasite DNA was extracted in a 96-well format as described by Zainabadi et al*.* [28]. The eluted DNA was then quantified by fluorometric quantification (Qubit, Thermo Fisher), adjusted to 20 ng/μL and stored at − 20 °C until further use. Amplicons from the target regions (exon 2 of the *hrp2* and *hrp3* genes) were generated using multiplexed nested PCR assays with indexed primers containing specific tags (barcodes)[29–31]. A total of 4 μL of PCR reactions from each sample were pooled (96 samples) to increase the sample volume and to minimize the use of samples for the downstream steps of the protocol. For each pool, amplicons were purified using AMPure XP beads (Beckman Coulter) according to the manufacturer's protocol. This was done to remove dNTPs, salts, primers, and primer dimers. The quality of the purified PCR products was assessed by analysing eluates containing the purified amplicons on a fragment analyser (Agilent). The concentration of DNA in the pooled fragments was assessed by fluorometric quantification (Qubit, Thermo Fisher). Pooled libraries were denatured with NaOH to a final concentration of 0.1N and diluted with hybridization buffer prior to sequencing. Sequencing was performed with MiSeq v2 reagents using the 300-cycle kit (Illumina) as recommended by the manufacturer. A phred score of 30 was used to demultiplex and quality trim the raw sequences. Primer sequences were trimmed from the 5′ end of the sequences to avoid any bias of the primers in the sequenced fragments. Base calling was performed by comparing the reads to a custom database consisting of the 3D7 reference sequence (v45). Bioinformatic analyses were performed using CLC Genomics Workbench22 (Qiagen). Laboratory reference parasite strains (Dd2 with a single *hrp2* deletion; 7G8; HB3 with a single *hrp3* deletion and 3601, a Cambodian culture-adapted parasite) were used as controls to validate each run.

### Ethical considerations

The protocol was approved and received authorisation n° 2022–05-117 from the Burkina Faso Health Research Ethics Committee. Before the start of the study, a community meeting was held in each selected village to discuss the study with community leaders. Individual informed consent was obtained from each head of household during a home visit before any study procedure.

### Statistical analysis

The socio-demographic characteristics of the participants were described using summary statistics. Continuous variables were described using the mean (and standard deviation), median (and IQR 25–75) and range. The one-way ANOVA and the Kruskal–Wallis tests were used to test differences in means and medians, respectively. The Mann–Whitney test was used to assess the distribution of parasite density in positive and negative RDT samples Categorical variables were presented as frequencies. Chi-square tests were used to assess differences in false-negative RDTs, *hrp2* deletion rates between sites as well as individual parameters, including gender (female and male). A p-value test of < 0.05 was considered statistically significant. Analysis was carried out using MedCalc® Statistical Software version 20.218 (MedCalc Software Ltd, Ostend, Belgium; https://www.medcalc.org; 2023).

## Results

### Baseline characteristics of the study participants

Of the 1,140 children recruited at the three study sites, 251 whose thick and thin blood smears were microscopically positive for *P. falciparum* were selected to be tested for the absence of the hrp2 and hrp3 genes. Baseline characteristics of the study population by site are summarized in Table 1. Microscopy parasite densities ranged from 16 to 544,000 parasites/μl and the median parasite density was 1,365 parasites/µl (IQR = 449–8210).

**Table 1:** Baseline characteristics of the study individual by site

	Site			All site	p-value
	Banfora	Gaoua	Orodara		
No. of patients	33	183	35	251	-
Males, %	45.5%	48.6%	45.7%	47.8%	0.9*
Age (years)					-
mean (SD*)	5.6 (2.2)	5.8 (2.5)	6.9 (1.5)	5.6 (2.4)	0.02**
range (min–max)	0.6–10	0.5–10	2.2–9.6	0.5–10	-
Temperature (°C), day 0					-
mean (SD*)	37.0 (0.6)	36.8 (0.6)	36.5 (0.5)	36.7 (0.6)	0.1**
Parasitaemia light microscopy(µl), day 0					-
median (IQR)	4831 (928–32,451)	1350 (461–6639)	706 (168–7019)	1365 (449–8210)	0.1***
range (min–max)	24–544,000	16–207,273	16–180,870	16–544,000	-

^*^Chi-squared test, **ANOVA (one-way), ***Kruskal–Wallis test

### HRP2-based RDT results

**Fig. 2:**
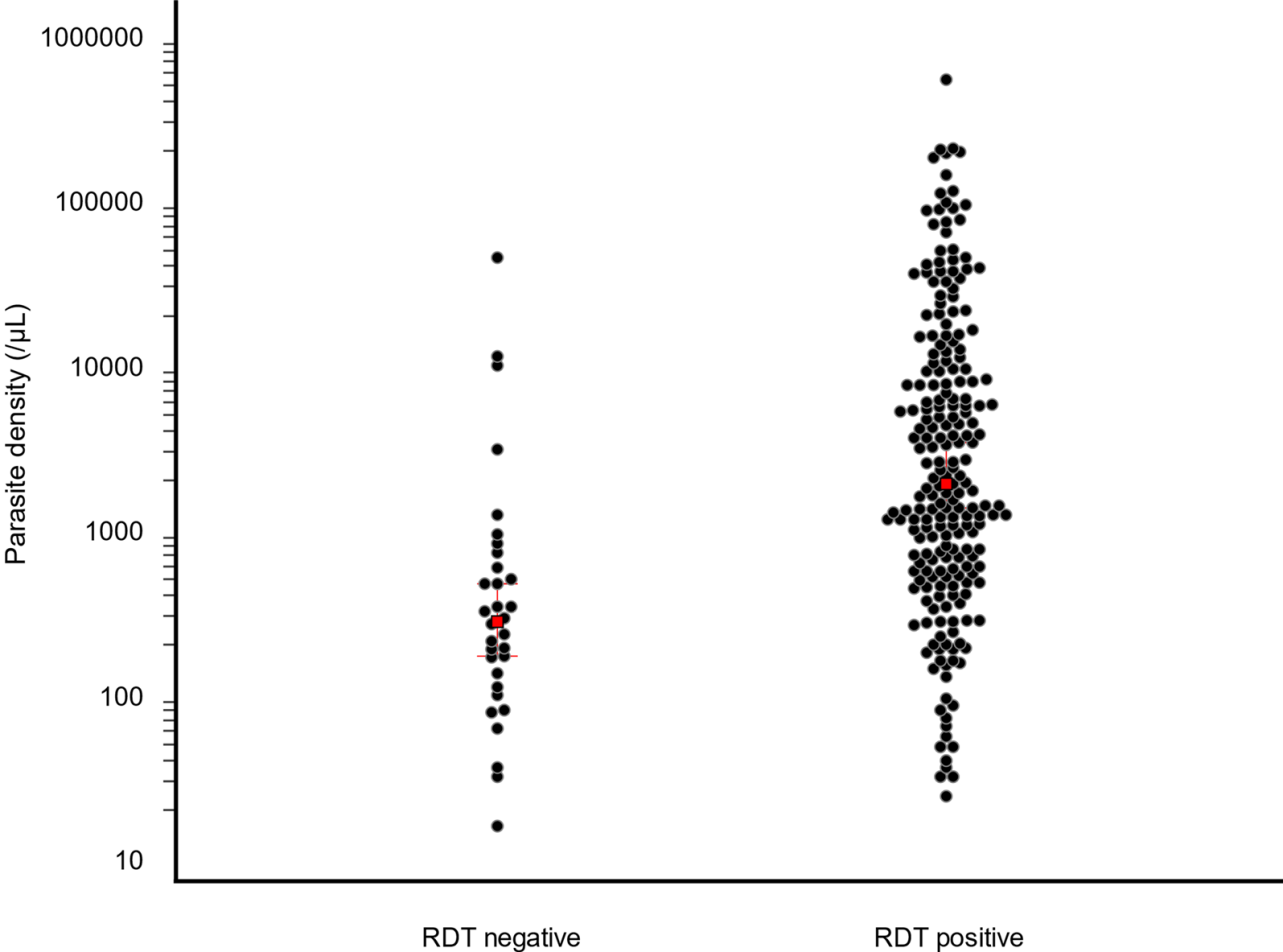
Distribution of the parasite density of the samples tested as positive and negative by HRP2-based RDT (red dots are median)

Of the 251 microscopy-positive *P. falciparum* isolates tested with the HRP2-based RDT, there were 219 positive results (87.3%). The proportion of samples with negative RDT results among the microscopy-positive samples was 12.7% (32/251). No significant difference in the proportion of negative RDT cases was observed between sites (Orodara, 5/35, 14.3%; Gaoua, 24/183, 13.1% and Banfora, 3/33, 9.1%, p = 0.8, chi-squared test). The median parasite density of the samples differed significantly between those tested positive (1910 parasites/µL, IQR = 565–10118) and those tested negative (279 parasites/µL, IQR = 123–661, p < 0.0001, Mann–Whitney test) (Fig. 2).

Of the 32 microscopy-positive *P. falciparum* isolates that were negative by HRP2-based RDT, 22 (69%) isolates had a parasite density < 500 µL and 27 (84%) isolates had a parasite density < 1000 µL. The parasite densities > 1000 parasites/µL of the five isolates that tested negative with HRP2-based RDT were 1240/µL, 3083/µL, 9951/µL, 11,320/µL and 44,757/µL.

### Detection *of hrp2/3* gene deletion

All extracted isolate DNA were successfully sequenced for the detection of the *hrp2* and *hrp3* gene deletions. The frequency distribution of *P. falciparum hrp2* and *hrp3* gene deletions in the study sites are presented in Table 2.

**Table 2:** Frequency distribution of *P. falciparum hrp2* and *hrp3* deletions

Characteristics	Sites					P-value
	N(%)	BANFORA	GAOUA	ORODARA	All site	
Samples	N	33	183	35	251	< 0.0001
	%	13.1%	72.9%	13.9%	100%	
RDT false negative	N	3	24	5	32	
	%	9.1%	13.1%	14.3%	12.7%	
RDT positive	N	30	159	30	219	
	%	12.0%	63.0%	12.0%	87.3%	
No deletion	N	32	177	31	240	< 0.0001
	%	97.0%	96.6%	88.6%	95.6%	
*hrp2* gene deletion	N	1	6	4	11	
	%	3.0%	3.3%	11.4%	4.4%	
*hrp3* gene deletion	N	0	0	0	0	
	%	0.0%	0.0%	0.0%	0.0%	
Dual *hrp2/hrp-3* gene deletion	N %	0 0.0%	0 0.0%	0 0.0%	0 0.0%	

RDT status in the study sites

A total of 95.6% (240/251) of the isolates presented parasites with no deletion for *hrp2* and *hrp3* genes while 4.4% (11/251) had a single *hrp2* deletion. No parasite had a single deletion of the *hrp3* gene or the dual deletion of the *hrp2/3* gene. The highest frequency of parasites with *hrp2* deletion was found in Orodara (4/35, 11.4%), followed by Gaoua (6/183, 3.3%) and Banfora (1/33, 3.0%) (Table 2).

### Relationship between HRP2-based RDT results and *hrp2* and *hrp3* gene deletions

Of the 32 microscopy-positive *P. falciparum* samples that tested negative with the HRP2-based RDT, only 2 (6.2%) had parasites with a single *hrp2* deletion and a parasite density of 32 and 134 parasites/µL, respectively. These 2 samples were likely missed by RDT due to their low parasitaemia. For the other samples (N = 30) that did not have the *pfhrp2/3* deletion but were also negative by the HRP2-based RDT (median parasite density of 279/µL IQR = 123–661), the false negative RDT results were probably due to the detection threshold of the test. For the 9 samples that had parasites with a single *hrp2* deletion and tested positive by HRP2-based RDT, the median parasite density was 471/µL (IQR = 202-1211).

## Discussion

PfHRP2-based RDTs remain a fully accessible diagnostic tool for the management of suspected malaria cases, particularly in areas where microscopy is not available. However, the recent emergence and spread of parasites lacking the *hrp2* and *hrp3* genes threatens to compromise patient care efforts and control programmes. False-negative RDTs due to *hrp2* and *hrp3* gene deletion have been reported in several studies from Africa [12, 16, 21, 32].

The prevalence of *P. falciparum* with *hrp2* gene deletion was low, estimated at 4.4% (11/251) of microscopy-positive samples. Overall, 12.7% (32/251) false negative RDT results among the samples tested with the HRP2-based RDT. Most of these samples had low parasite densities. Only two samples with false negative RDT results had also a single *hrp2* deletion. These false-negative RDT results were probably not related to the *hrp2* deletion but rather to their low parasitaemia, below the detection threshold of the HRP2-based RDT. Similarly, 30 other samples had false-negative results that appeared to be related to detection limits. However, others factors than *hrp2/3* genes deletions, including operator error, lot-to-lot RDT variability, and infection with non-falciparum species are the most common causes of false-negative HRP2-based RDT results in Africa [33, 34]. Nine isolates that had parasites with a single *hrp2* deletion but tested positive with the HRP2-based RDT were also found. Positive results observed with the PfHRP2-based RDTs were due to parasite densities above the detection limit and the expression of the HRP3 protein, which is known to cross-react with monoclonal antibodies against the HRP2 protein.

Although this study is the first study conducted in Burkina Faso, previous studies have been conducted in neighbouring countries, including Ghana, Mali, Senegal and Nigeria. As shown in these studies, the data from this study confirm that parasites with a single *pfhrp2* deletion are circulating at low rates in the region [18, 20, 21]. In fact, in these countries, varying low proportions of parasites with a single *hrp2* deletion have been shown, far from those found in clinical cases in some hospitals in Eritrea which reach up to 80% [12]. There are geographical differences in the prevalence of deletions in areas where malaria is endemic. Studies carried out in Ghana have not observed any deletions in the middle belt of Ghana [32], whereas deletions have been observed in Ghanaian isolates in the coastal part of Ghana [35], with worrying proportions of parasites with a double deletion in 30.7 and 17.2% of isolates for the *pfhrp2* and *pfhrp3* genes, respectively [35].

Of note, the study presented here has some limitations. First, the samples tested for *hrp2/3* deletions were obtained from a cross-sectional household survey of asymptomatic children and was nested within a larger research project that aimed at investigating the prevalence of asymptomatic malaria using rapid diagnostic tests. As a result, the population tested differs from the target population described in the WHO standard survey protocol who are individuals seeking care for febrile illness at health. Second, the selected 251 microscopy-positive *P. falciparum* samples used to test for *hrp2/3* deletions were not confirmed by PCR. For the 5 RDT-negative cases with parasite densities > 1000 parasites/uL (1240/µL, 3083/µL, 9951/µL, 11,320/µL and 44,757/µL) and no gene deletions, it cannot be excluded that these infections were due to non-*P. falciparum* species or to mixing with non-*P. falciparum* species.

Finally, the methodology used was only able to detect monoclonal infections with *hrp2-*deleted parasites and polyclonal infections where all clones had *hrp2*-deleted parasites. Previous research has shown an interaction between the multiplicity of infections (MOI) and the *hrp2* and *hrp3* deletions in malaria-endemic areas [36]. Polyclonal infections could mask the deletions and lead to an underestimation of the prevalence of deletions. The frequency of deletion among these polyclonal infections is higher than that of monoclonal infections [36]. The results obtained in this study showed a low prevalence of deletion and this could be caused by the existence of MOI as well as mixed with deleted and non-deleted parasites classified as non-deleted in this study.

## Conclusion

The results of this study show that *P. falciparum* isolates lacking the *hrp2* gene are present but at low levels in Burkina Faso. It is reassuring to note that parasites with a single deletion of the *hrp2* gene do not appear to reduce the performance of HRP2-based RDTs. However, given the geographically heterogeneous prevalence of gene deletion, ongoing monitoring is necessary, as the situation could change rapidly.

## Data Availability

All data generated or analysed during this study are available from the corresponding author and can be provided if required.

## References

[1] Annuaire Statistique 2021. Ministère de la Santé et de l'Hygiène Publique du Burkina Faso 2021.

[2] WHO (2015). Guidelines for the treatment of malaria.

[3] Wongsrichanalai C, Barcus MJ, Muth S, Sutamihardja A, Wernsdorfer WH (2007). A review of malaria diagnostic tools: microscopy and rapid diagnostic test (RDT). Am J Trop Med Hyg.

[4] Ohrt C, Purnono M, Sutamihardja A, Tang D, Kain KC (2002). Impact of microscopy error on estimates of protective efficacy in malaria-prevention trials. J Infect Dis.

[5] Moody A (2002). Rapid Diagnostic tests for malaria parasites. Clin Microbiol Rev.

[6] Cunningham J, Jones S, Gatton ML, Barnwell JW, Cheng Q, Chiodini PL (2019). A review of the WHO malaria rapid diagnostic test product testing programme (2008–2018): performance, procurement and policy. Malar J.

[7] Murray CK, Gasser RA, Magill AJ, Miller RS (2008). Update on rapid diagnostic testing for malaria. Clin Microbiol Rev.

[8] Wellems TE, Howard RJ (1986). Homologous genes encode two distinct histidine-rich proteins in a cloned isolate of *Plasmodium falciparum*. Proc Natl Acad Sci USA.

[9] Rock EP, Marsh K, Saul AJ, Wellems TE, Taylor DW, Maloy WL (1987). Comparative analysis of the *Plasmodium falciparum* histidine-rich proteins HRP-I, HRP-II and HRP-III in malaria parasites of diverse origin. Parasitology.

[10] Kong A, Wilson SA, Ah Y, Nace D, Rogier E, Aidoo M (2021). HRP2 and HRP3 cross-reactivity and implications for HRP2-based RDT use in regions with *Plasmodium falciparum hrp2* gene deletions. Malar J.

[11] Boyce MR, O’Meara WP (2017). Use of malaria RDTs in various health contexts across sub-Saharan Africa: a systematic review. BMC Public Health.

[12] Berhane A, Anderson K, Mihreteab S, Gresty K, Rogier E, Mohamed S (2018). Major threat to malaria control programs by *Plasmodium falciparum* lacking histidine-rich protein 2. Eritrea Emerg Infect Dis.

[13] Gamboa D, Ho MF, Bendezu J, Torres K, Chiodini PL, Barnwell JW (2010). A large proportion of P falciparum isolates in the Amazon region of Peru lack pfhrp2 and pfhrp3: implications for malaria rapid diagnostic tests. PLoS ONE.

[14] Dorado EJ, Okoth SA, Montenegro LM, Diaz G, Barnwell JW, Udhayakumar V (2016). Genetic characterisation of *Plasmodium falciparum* isolates with deletion of the pfhrp2 and/or pfhrp3 genes in Colombia: the Amazon region, a challenge for malaria diagnosis and control. PLoS ONE.

[15] Pati P, Dhangadamajhi G, Bal M, Ranjit M (2018). High proportions of pfhrp2 gene deletion and performance of HRP2-based rapid diagnostic test in *Plasmodium falciparum* field isolates of Odisha. Malar J.

[16] Berhane A, Russom M, Bahta I, Hagos F, Ghirmai M, Uqubay S (2017). Rapid diagnostic tests failing to detect *Plasmodium falciparum* infections in Eritrea: an investigation of reported false negative RDT results. Malar J.

[17] Kozycki CT, Umulisa N, Rulisa S, Mwikarago EI, Musabyimana JP, Habimana JP (2017). False-negative malaria rapid diagnostic tests in Rwanda: impact of *Plasmodium falciparum* isolates lacking *hrp2* and declining malaria transmission. Malar J.

[18] Koita OA, Doumbo OK, Ouattara A, Tall LK, Konaré A, Diakité M (2012). False-negative rapid diagnostic tests for malaria and deletion of the histidine-rich repeat region of the *hrp2* gene. Am J Trop Med Hyg.

[19] Parr JB, Verity R, Doctor SM, Janko M, Carey-Ewend K, Turman BJ (2017). Pfhrp2-deleted *Plasmodium falciparum* parasites in the Democratic Republic of the Congo: a national cross-sectional survey. J Infect Dis.

[20] Amoah LE, Abankwa J, Oppong A (2016). *Plasmodium falciparum* histidine rich protein-2 diversity and the implications for PfHRP 2: based malaria rapid diagnostic tests in Ghana. Malar J.

[21] Funwei R, Nderu D, Nguetse CN, Thomas BN, Falade CO, Velavan TP (2019). Molecular surveillance of pfhrp2 and pfhrp3 genes deletion in *Plasmodium falciparum* isolates and the implications for rapid diagnostic tests in Nigeria. Acta Trop.

[22] Agaba BB, Yeka A, Nsobya S, Arinaitwe E, Nankabirwa J, Opigo J (2019). Systematic review of the status of pfhrp2 and pfhrp3 gene deletion, approaches and methods used for its estimation and reporting in *Plasmodium falciparum* populations in Africa: review of published studies 2010–2019. Malar J.

[23] Berhane A, Anderson K, Mihreteab S, Gresty K, Rogier E, Mohamed S (2018). Major Threat to malaria control programs by *Plasmodium falciparum* lacking histidine-rich protein 2 Eritrea. Emerg Infect Dis.

[24] WHO (2018). Protocol for estimating the prevalence of pfhrp2/pfhrp3 gene deletions among symptomatic falciparum patients with false-negative RDT results.

[25] Soma DD, Zogo BM, Somé A, Tchiekoi BN, de Hien DF, S, Pooda HS,  (2020). *Anopheles* bionomics, insecticide resistance and malaria transmission in southwest Burkina Faso: a pre-intervention study. PLoS ONE.

[26] Epopa PS, Collins CM, North A, Millogo AA, Benedict MQ, Tripet F (2019). Seasonal malaria vector and transmission dynamics in western Burkina Faso. Malar J.

[27] Ben-Shlomo Y, Brookes ST, Hickman M. Epidemiology, evidence-based medicine and public health. 6th Edn. Wiley-Blackwell, 2013.

[28] Zainabadi K, Nyunt MM, Plowe CV (2019). An improved nucleic acid extraction method from dried blood spots for amplification of *Plasmodium falciparum kelch13* for detection of artemisinin resistance. Malar J.

[29] Issa MS, Warsame M, Mahamat MHT, Saleh IDM, Boulotigam K, Djimrassengar H (2023). Therapeutic efficacy of artesunate–amodiaquine and artemether–lumefantrine for the treatment of uncomplicated falciparum malaria in Chad: clinical and genetic surveillance. Malar J.

[30] Mihreteab S, Platon L, Berhane A, Stokes BH, Warsame M, Campagne P (2023). Increasing prevalence of artemisinin-resistant HRP2-negative malaria in Eritrea. N Engl J Med.

[31] L’Episcopia M, Doderer-Lang C, Perrotti E, Priuli GB, Cavallari S, Guidetti C (2023). Polymorphism analysis of drug resistance markers in *Plasmodium falciparum* isolates from Benin. Acta Trop.

[32] Addai-Mensah O, Dinko B, Noagbe M, Ameke SL, Annani-Akollor ME, Owiredu EW (2020). *Plasmodium falciparum* histidine-rich protein 2 diversity in Ghana. Malar J.

[33] Watson OJ, Sumner KM, Janko M, Goel V, Winskill P, Slater HC (2019). False-negative malaria rapid diagnostic test results and their impact on community-based malaria surveys in sub-Saharan Africa. BMJ Glob Health.

[34] Wu L, van den Hoogen LL, Slater H, Walker PGT, Ghani AC, Drakeley CJ (2015). Comparison of diagnostics for the detection of asymptomatic *Plasmodium falciparum* infections to inform control and elimination strategies. Nature.

[35] Duah-Quashie NO, Opoku-Agyeman P, Bruku S, Adams T, Tandoh KZ, Ennuson NA (2022). Genetic deletions and high diversity of *Plasmodium falciparum* histidine-rich proteins 2 and 3 genes in parasite populations in Ghana. Front Epidemiol.

[36] Molina-de la Fuente I, Benito MJS, Flevaud L, Ousley J, Pasquale HA, Julla A (2023). *Plasmodium falciparum pfhrp2* and *pfhrp3* gene deletions in malaria-hyperendemic region South Sudan. Emerg Infect Dis.

